# Evaluation of the Cutaneous Immunological Milieu Before and After Treatment With Meglumine Antimoniate in Dogs Naturally Affected by Leishmaniosis due to *Leishmania infantum*

**DOI:** 10.1111/vde.70056

**Published:** 2026-02-18

**Authors:** M. Platenik, A. Di Loria, L. Archer, M. Saridomichelakis, A. S. Benvenuto, D. Santoro

**Affiliations:** ^1^ Department of Small Animal Clinical Sciences, College of Veterinary Medicine University of Florida Gainesville Florida USA; ^2^ Department of Veterinary Medicine and Animal Productions University “Federico II” Napoli Italy; ^3^ Clinic of Medicine, Faculty of Veterinary Science University of Thessaly Karditsa Greece; ^4^ Temesa Veterinary Medical Center Amantea Italy

**Keywords:** canine, cutaneous, immune responses, leishmaniosis, meglumine antimoniate

## Abstract

**Background:**

Canine leishmaniosis (CanL) is a zoonotic disease of variable severity. The typical immune response is driven by an exaggerated humoral immune response. Protective immunity is mediated by pro‐inflammatory cytokines that enhance macrophage leishmanicidal activity.

**Objective:**

To evaluate the cutaneous and the systemic immune responses as well as the cutaneous parasitic load in affected dogs before and after 28 days of treatment with meglumine antimoniate.

**Animals:**

Twelve dogs with CanL and skin lesions, treated with meglumine antimoniate at a target dose of 100 mg/kg subcutaneously every 24 h, were prospectively enrolled.

**Methods and Materials:**

Before and after 28 days of treatments, blood samples and skin biopsies were collected. Circulating levels of host defence peptides, leptin and cytokines were determined via enzyme‐linked immunosorbent assay (ELISA). Paraffin‐embedded skin biopsies were processed for routine immunofluorescence and positive cells were identified using commercially available anti‐canine antibodies. Parasitic load also was determined via molecular methods. All variables were statistically analysed with the significance level set at 0.05.

**Results:**

Among the cutaneous cell types investigated, there was a decrease in the number of T‐box transcription factor TBX21 (Tbet^+^) (*p* = 0.016), GATA binding protein 3 (GATA3^+^) (*p* = 0.016), interleukin (IL)‐17A^+^ (*p* = 0.03) cells and neutrophils (*p* = 0.008) after treatment, whereas there were no significant changes in forkhead box protein P3 (FoxP3^+^) regulatory T and ionised calcium‐binding adaptor molecule 1 (Iba1^+^) cells. A lack of change in serum concentration of inflammatory mediators was found. Finally, cutaneous parasitic load was significantly lower after treatment (*p* = 0.03).

**Conclusions and Clinical Relevance:**

The results of this study show that the reduction of cutaneous parasitic load after meglumine antimoniate treatment results in downregulation of innate and adaptive cutaneous inflammatory responses.

## Graphical abstract

Background: Canine leishmaniosis (CanL) is a zoonotic disease of variable severity. The typical immune response is driven by an exaggerated humoral immune response. Protective immunity is mediated by pro‐inflammatory cytokines that enhance macrophage leishmanicidal activity. Objective: To evaluate the cutaneous and the systemic immune responses as well as the cutaneous parasitic load in affected dogs before and after 28 days of treatment with meglumine antimoniate. Conclusions and Clinical Relevance: The results of this study show that the reduction of cutaneous parasitic load after meglumine antimoniate treatment results in downregulation of innate and adaptive cutaneous inflammatory responses.

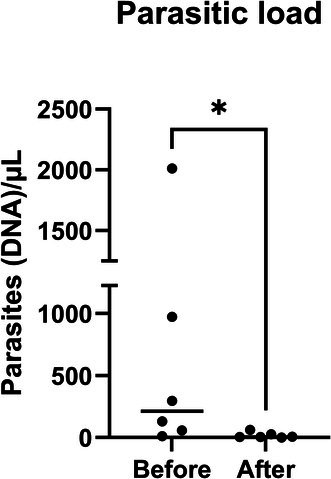

## 1 Introduction

Canine leishmaniosis (CanL) caused by *Leishmania infantum* (a.k.a. *L. chagasi*) is an important zoonotic disease, that can be severe or even fatal, and it is endemic in Mediterranean basin, Middle East and South America [1]. Also, cases have emerged in USA and Canada, where transmission is thought to be mainly transplacental, rather than through sand fly bites [2]. The cutaneous and systemic clinical manifestations of CanL result from granulomatous or pyogranulomatous inflammation, and the deposition of immune complexes [1]. Because of the diversified host–parasite relationship, CanL is characterised by variable clinical, clinicopathological and histological manifestations, parasitic densities, and immunological profiles of inflammatory cells [1].

Little is known about the cutaneous and systemic immunologic response present in CanL and multiple factors may affect the differentiation of newly activated T‐helper (Th) cells into mature Th1 or Th2 cells. One of these factors may be the cytokine milieu surrounding the newly activated T cells [3]. In particular, Th1 cytokines are necessary for macrophage activation and killing of the parasite, while Th2 cytokines have been associated with an exaggerated yet unprotective humoral immune response [3, 4]. Also, immunoregulatory cytokines mainly produced by T‐regulatory cells (Tregs) [5, 6], such as interleukin (IL)‐10 and transforming growth factor (TGF)‐β, compromise antigen presentation and inhibit T‐cell co‐stimulation and proliferation [5]. They also block macrophage activation and production of Th1 cytokines [5]. High levels of such immunoregulatory cytokines have been associated with marked humoral immune responses, reduced cell‐mediated immunity and high parasite burden [6].

Information on innate immunity during CanL is sparse, despite neutrophils and macrophages being known as the first line of defence against *Leishmania* organisms [7]. Additionally, the in vitro efficacy of natural and synthetic host defence peptides (HDPs) against *Leishmania* parasites [8, 9], suggests a possible role in the pathogenesis of CanL. Natural HDPs, such as defensins, cathelicidins and S100A proteins, are small microbiologically and immunologically active proteins produced and secreted by epithelial and immune cells [10, 11]. Defensins and S100A proteins exhibit a wide variety of anti‐microbial—including anti‐*Leishmania*—effects by enhancing the activity of immune cells and helping to control the infection [8]. Likewise, cathelicidins can promote inflammatory response and enhance phagocytosis [8, 10]. Another mediator important in the innate and adaptive immune responses is leptin. It is a 16 kDa hormone that is secreted primarily by the adipose tissue, and also by the stomach, skeletal muscles, placenta, memory T cells and macrophages [12, 13]. Leptin, together with IL‐1 and IL‐6, can act as an early acute‐phase reactant, and its production can be upregulated by other inflammatory mediators, such as tumour necrosis factor (TNF)‐α and IL‐1 [13, 14].

The treatment of choice for CanL includes short‐term use of meglumine antimoniate or miltefosine in combination with long‐term allopurinol administration [15]. Although the exact mechanism‐of‐action of meglumine antimoniate has not been completely established, it is hypothesised that it inhibits the glycolytic activity and the oxidation of fatty acids in *Leishmania* amastigotes, leading to parasite death [16]. Also, in vitro studies show that meglumine antimoniate can enhance phagocytosis by macrophages [17] and TNF‐α production by peripheral blood mononuclear cells [18].

In order to increase our knowledge on the immunological changes induced by meglumine antimoniate treatment of CanL, the aims of this study were to compare the populations of cutaneous immune cells (FoxP3+ [forkhead box protein P3], GATA‐3+ [GATA binding protein 3], Iba1+ [ionised calcium‐binding adaptor molecule 1], IL‐17A+, T‐bet+ [T‐box transcription factor TBX21] and neutrophil elastase+) via indirect immunofluorescence (IIF), and the parasitic load via quantitative (q)PCR, before and after 28 days of treatment with this drug. Circulating levels of leptin, selected cytokines (IL‐4, IL‐10, IL‐17A, interferon [IFN]‐γ and TGF‐β) and host defence peptides (canine beta defensin [cBD]3‐like, canine cathelicidin [cCath] and S100A8 protein) were also assessed.

## 2 Materials and Methods

### 2.1 Ethical Consent

This was a prospective study performed using skin biopsy and serum samples. The use of dogs was in accordance with the European Communities Council Directive 86/609/EEC and Greek laws (1197/81 and 2015/92), including the owner consent form, and the experimental protocol had been approved by State authorities (licence no. EL41BIO‐02/2204/4‐6‐09).

### 2.2 Dogs

Twelve dogs with CanL and skin lesions, treated with meglumine antimoniate (Glucantime; Boehringer Ingelheim Animal Health) at a target dose of 100 mg/kg (actual dose 99.48 ± 0.97 mg/kg) subcutaneously every 24 h, were prospectively enrolled. All dogs presented clinical signs and clinicopathological abnormalities of the disease (see Table S1 in Supporting Information), and the diagnosis was confirmed by the detection of amastigotes in stained lymph node and/or bone marrow aspiration smears, and by positive serology (indirect fluorescent antibody test with a cut‐off titre: ≥ 1:160) [17]. According to LeishVet staging system, the 12 dogs were classified in stage II (11) or stage III (1) [18, 19, 20]. Administration of topical, systemic and deposit corticosteroids for the previous 2, 4 and 8 weeks, respectively, recent use of other anti‐inflammatory or immunosuppressive medications, and previous treatment for CanL were exclusion criteria.

### 2.3 Skin Biopsy Collection

Skin biopsies were collected with 8 mm sterile biopsy punches under local anaesthesia (lidocaine 2%, Xylocaine injection; Astra). The samples were obtained from skin with macroscopic lesions of exfoliative and ulcerative dermatitis before treatment and from approximately the same location after treatment. Each biopsy sample was thoroughly blotted onto a sterile gauze to remove excess blood and cut with a sterile scalpel blade perpendicularly to the skin surface into two pieces. One of them (flash‐frozen) was used for real‐time qPCR, while the second one was fixed in 10% buffered formalin for ≤ 48 h, embedded in paraffin, and used for IIF.

### 2.4 Cutaneous Indirect Immunofluorescence

For immunostaining, 3‐μm‐thick sections were processed using the immunohistochemical polymer procedure as described previously [20]. Briefly, the slides were blocked using a casein solution (Power Block; BioGenex) followed by an extra blocking step with normal donkey serum (BioGenex). Blocking serum was removed, and the sections were incubated overnight with primary anti‐canine antibodies (Table 1), all at 1:200 dilution based on manufacturer's recommendations and previous studies [21, 22, 23]. After washing, they were incubated for 60 min with 1:1000 dilution of fluorochrome‐labelled secondary antibodies (Alexa; Invitrogen) specific for each primary antibody (Table 1), according to the manufacturer's recommendations. Finally, DAPI (4′,6‐diamidino‐2‐phenylindole) (Invitrogen) was used as a counterstain for nuclear detection. The slides were mounted using Vectashield Mounting Medium (Vector Laboratories) and were examined under an inverted fluorescent microscope (Evos FL cell imaging system; Life Technologies). Up to 300 cells were counted in the entire section, from the left edge to the right edge of the sample (epidermis, dermis and subcutis) and the average cytoplasmic fluorescence signal was recorded using a computer imaging program (imagej 1.41; https://imagej.net/ij/).

**TABLE 1:** List of primary and secondary antibodies.

Primary antibody	Type	Catalogue no.	Secondary antibody
T‐bet	Polyclonal rabbit	LS‐C29739	Alexa Fluor 568 donkey anti‐rabbit
GATA3	Polyclonal goat	ab113519	Alexa Fluor 594 donkey anti‐goat
IL‐17A	Polyclonal rabbit	ab79056	Alexa Fluor 568 donkey anti‐rabbit
FoxP3	Polyclonal rabbit	sab2108477	Alexa Fluor 568 donkey anti‐rabbit
Iba1	Monoclonal mouse	MABN92	Alexa Fluor 488 donkey anti‐mouse
Neutrophil elastase	Polyclonal rabbit	ab68672	Alexa Fluor 568 donkey anti‐rabbit

Abbreviations: FoxP3, forkhead box protein P3; GATA‐3, GATA binding protein 3; Iba1, ionised calcium‐binding adapter molecule 1; IL, interleukin; T‐bet, T‐box transcription factor TBX21.

### 2.5 Circulating HDPs, Leptin and Cytokines

A custom‐made competitive inhibition enzyme‐linked immunosorbent assay ([ci] ELISA) was used to measure serum concentrations of cBD3‐like and cCath [12, 13, 21, 22, 23, 24], and a commercially available ELISA kit (Antibodies‐online) was used to measure S100A8 following the manufacturers' recommendations.

A commercially available ELISA kit (EMD Millipore) was used to measure serum concentrations of canine leptin, following the manufacturer's recommendations.

Commercially available ELISA kits were used to measure serum concentrations of canine IL‐4, IL‐10, IL‐17A, IFN‐γ, and TGF‐β following the manufacturers' recommendations (R&D Systems).

### 2.6 Quantification of Parasitic Density

The quantification of *L. infantum* was performed via qPCR, using the PCR Advanced Kit for *L. infantum* and *L. donovani* (Genesig Primer Design; Usbio), according to the manufacturer's instructions and using the provided standardised samples with 100 ng/μL of DNA. The temperature profile consisted of an initial denaturation step at 95°C for 5 min, followed by 50 cycles of 3 s at 95°C and 30 s at 60°C. Quantification was done by means of a graded scale. All skin biopsy samples were run in duplicates and the amount of target DNA was normalised with respect to the endogenous control (housekeeping) genes. The specificity of PCR products was confirmed by single peak dissociation curves and results were expressed as number of parasites (copy number) per μL (user manual).

### 2.7 Statistical Analysis

Distribution of data was tested using the Shapiro–Wilks test. Results of IIF in the skin, serum concentrations of HDPs, leptin and cytokines, and cutaneous parasitic density before and after treatment were compared by paired Student's *t*‐test (if normally distributed) or Wilcoxon rank test (if not normally distributed); *p*‐values ≤ 0.05 were considered significant. All statistical comparisons were performed using prism10 (GraphPad Software Inc.).

## 3 Results

### 3.1 Response to Treatment

For all 12 dogs, the improvement of clinical signs and clinicopathological abnormalities at the end of the treatment period was considered satisfactory and comparable to that described in randomised controlled trials on meglumine antimoniate monotherapy in CanL [16, 20, 21, 22, 23, 24, 25, 26, 27].

### 3.2 Cutaneous Indirect Immunofluorescence

**FIGURE 1:**
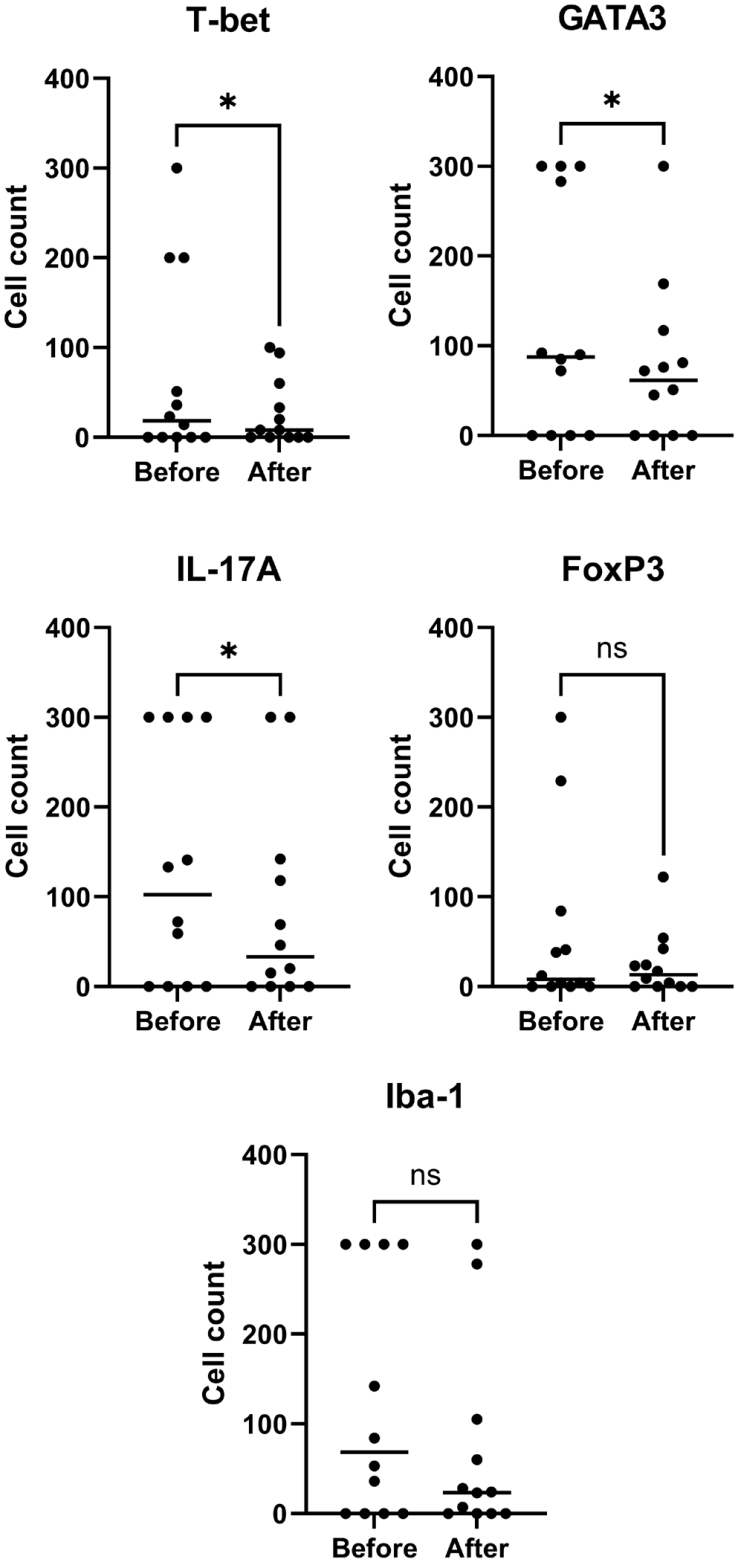
Median counts of cells positive for T‐box transcription factor TBX21 (T‐bet), GATA binding protein 3 (GATA3), interleukin (IL)17‐A, forkhead box protein P3 (FoxP3), ionised calcium‐binding adaptor molecule 1 (Iba‐1), and neutrophil elastase before and after 28 days of treatment with meglumine antimoniate (*n* = 12). Dots represent individual data points and horizontal lines indicate the median values (ns, nonsignificant difference; Significance: *p < 0.05; **p < 0.01).

There was a significant decrease in the number of T‐bet^+^ (*p* = 0.016), GATA3^+^ (*p* = 0.016), IL‐17A^+^ (*p* = 0.03), and neutrophil elastase^+^ (*p* = 0.008) cells after treatment. However, there was no significant change in the number of FoxP3^+^ (*p* = 0.08) and Iba1^+^ (*p* = 0.16) cells (Figure 1; Table S1).

### 3.3 Circulating HDPs, Leptin and Cytokines

**FIGURE 2:**
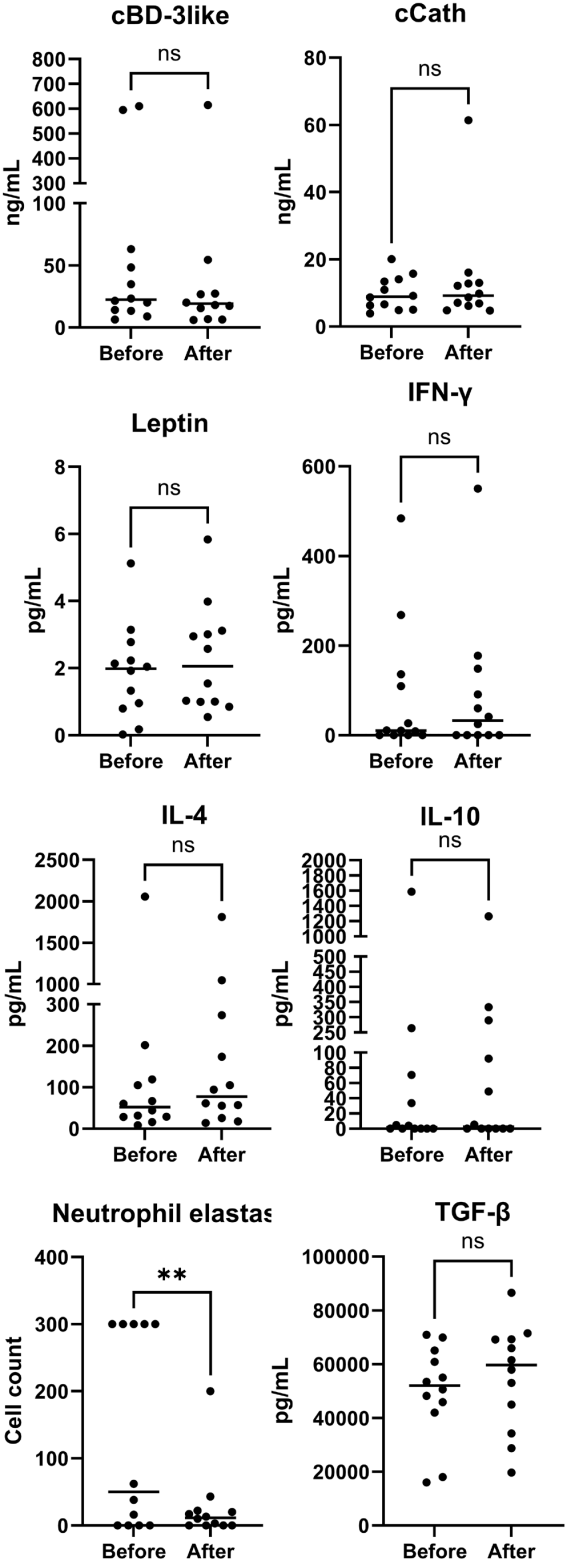
Concentrations of host defence peptides, leptin and cytokines in the serum before and after 28 days of treatment with meglumine antimoniate (*n* = 12). Dots represent individual data points and horizontal lines indicate the median values (ns, nonsignificant difference). cBD3‐like, canine β‐defensin 3‐like; cCath, canine cathelicidin; IFN, interferon; IL, interleukin; TGF, transforming growth factor.

There was no significant change in serum concentrations of leptin, cBD3‐like, cCath, IL‐4, IL‐10, IFN‐γ, or TGF‐β after treatment (Figure 2; Table S2). In most dogs, serum concentrations of S100A8 and IL‐17A were below detection levels and thus they were not further analysed.

### 3.4 Parasitic Density in the Skin

**FIGURE 3:**
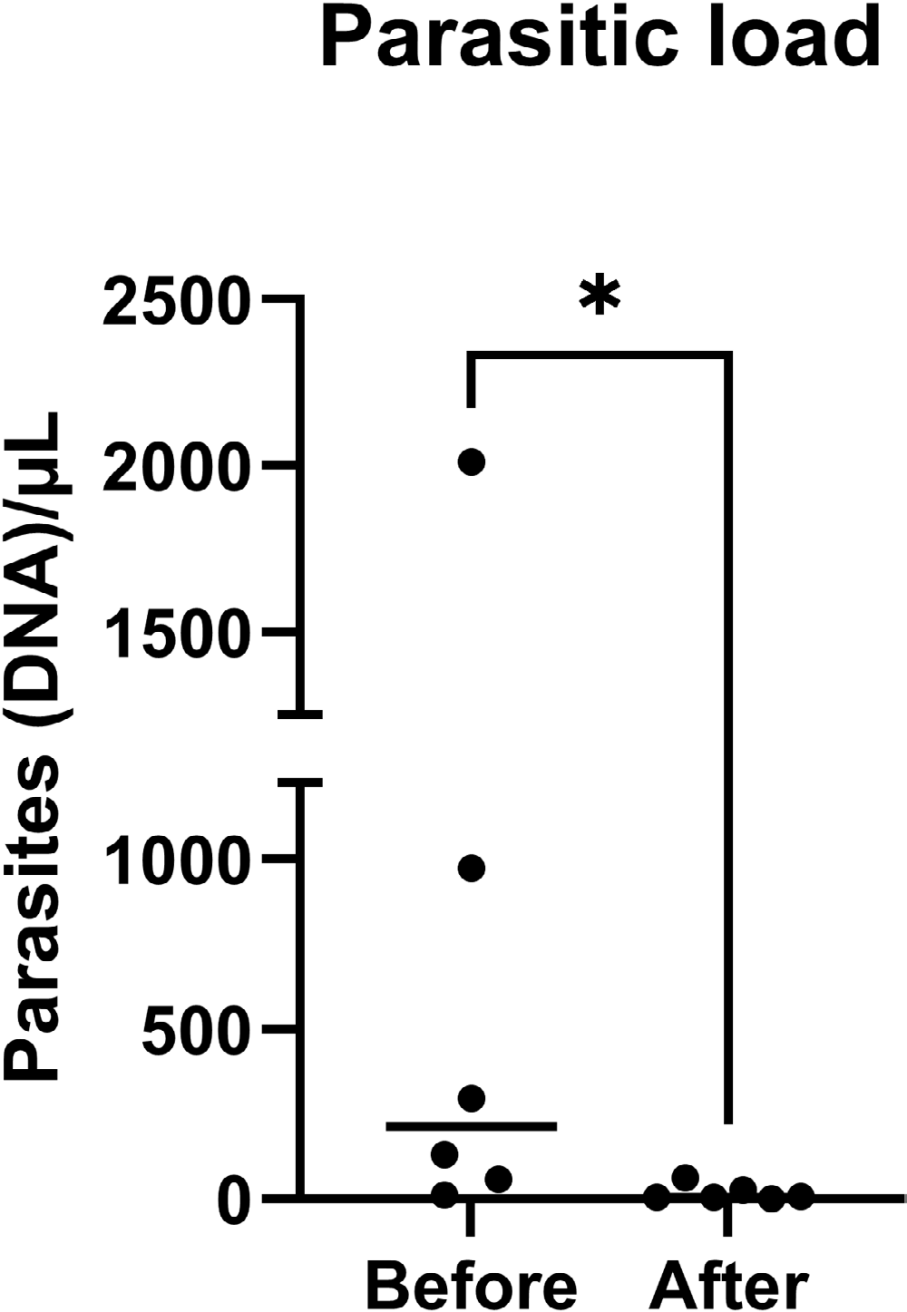
Quantitative PCR results of *Leishmania* density in the skin before and after 28 days of treatment with meglumine antimoniate (*n* = 6). Dots represent individual data points, and horizontal lines indicate the median values. (Significance: *p < 0.05).

Cutaneous parasitic load was significantly lower after treatment (*p* = 0.03) with a median load of 214 (13–2012) parasites/mL before and 6 (2–63) parasites/mL after treatment (Figure 3; Table S3).

## 4 Discussion

The results of this study show that the number of T‐bet+, GATA3+, IL‐17A+ cells and neutrophils in the skin decreased, in parallel with the number of parasites, after treatment with meglumine. However, no significant changes were found in the numbers of FoxP3+ and Iba1+ cells. Likewise, a lack of significant changes in serum levels of leptin, IL‐4, IL‐10, IFN‐γ, TGF‐β, cBD3‐like and cCath was seen. To the best of our knowledge, this is the first study investigating cutaneous and systemic immunological changes in CanL before and after treatment with meglumine antimoniate.

T‐bet is a T‐box transcription factor associated with type 1 immune responses leading to the release of cytokines that support cell‐mediated immunity, including IL‐2, IL‐12, TNF‐α and especially IFN‐γ. It is expressed mainly by CD4+ Th1 cells, and also by some CD8+, CD4‐/CD8‐ and natural killer cells. Tbet+ cells in the skin were significantly decreased after treatment, in parallel with the overall clinical improvement and—especially—with the reduction of cutaneous parasitic load. This finding indirectly supports the importance of the type 1 immunity for the eradication of *L. infantum* [8, 28, 29, 30, 31], because the lower the number of parasites in the skin, the less the need for Tbet+ cells to migrate to the skin to control them. However, this change was not accompanied by a decrease of IFN‐γ concentration in serum. On the contrary, the median serum concentration of IFN‐γ was nonsignificantly higher at the end of the 28 day treatment period. It is logical to assume that cutaneous expression of T‐bet and systemic IFN‐γ concentrations diverge owing to the production of the latter by Tbet+ cells in other organs and tissues. Furthermore, sustained serum concentrations of IFN‐γ during and after treatment have been proposed as a surrogate marker of therapeutic efficacy with restoration of parasite‐specific cell‐mediated immunity and controlled parasitic burden [32, 33].

The transcription factor GATA3 is mainly expressed by CD4+ Th2 cells, and also by CD8+, CD4‐/CD8‐ and natural killer cells. GATA3 has been associated with type 2 immune responses which promote susceptibility to *L. infantum* and culminate in the appearance of CanL. Upregulated GATA3 expression has been identified in the lesional (exfoliative dermatitis) skin of dogs with the disease [20, 21, 22, 23, 24, 25], and our results imply an inverse relationship between type 2 responses in the skin (i.e., reduced GATA3‐positive cells) and clinical improvement with reduction of local parasitic load. Interestingly, there was no change in the circulating levels of IL‐4. This result was not unexpected considering that effective treatment of CanL with liposome‐encapsulated meglumine antimoniate resulted, after 90 days, in reduction of blood CD4+ and not of CD8+ cells producing IL‐4 [31, 34]. Likewise, serum IL‐4 expression decreased only after 6 months in dogs treated with miltefosine–allopurinol in combination [30, 33].

The findings of the present study revealed a significant reduction in the presence of IL‐17A^+^ cells in the skin after treatment. These cells are present in increased numbers in the lesional skin of dogs with CanL [21], and they are the main source of IL‐17A, a pro‐inflammatory cytokine crucial for host defence against pathogens, as it facilitates the recruitment of neutrophils, macrophages and other lymphocytes, thereby bridging innate and adaptive immune responses. Additionally, IL‐17A activates canine keratinocytes to produce several pro‐inflammatory cytokines and HDPs, thus amplifying the inflammatory response. It can indirectly enhance Th1‐related responses and support parasite defence mechanisms by increasing nitric oxide production in macrophages [32, 35, 36]. Additionally, IL‐17A can promote the development of a Th2 response under certain conditions of mixed immune polarisation (e.g., chronic inflammation, infections and allergic diseases) [33]. In such conditions, IL‐17A can modulate the balance between Th1 and Th2 responses indirectly by influencing cytokine production and immune cell recruitment. Unfortunately, circulating levels of IL‐17A were not detectable in the present cohort, not allowing us to study the correlation between cutaneous infiltration of Th17 cells and systemic production of their main cytokine.

The findings of the current study reveal that along with the decrease in Th2 (GATA3^+^) cells, there is a decrease in Th1 (T‐bet^+^) and Th17 (IL‐17A^+^) cells in the skin of dogs with CanL after treatment with meglumine antimoniate. This is probably the result of reduced parasitic density and the ensuing downregulation of inflammatory cell infiltration, without an obvious shift in T‐helper immunophenotype. Nevertheless, the decreased number of all these inflammatory cells is reflected in the improvement of macroscopic skin lesions [24].

The Iba‐1 is a specific marker for the monocyte/macrophage lineage, and is expressed by macrophages and dendritic cells [21]. Macrophages are abundant in both lesional and normal‐looking skin of dogs with leishmaniosis [21, 24, 25, 26, 27, 28, 33] and, along with the dendritic cells, are considered to be of major importance in the pathogenesis of the disease. In this context, macrophages and dendritic cells serve as antigen‐presenting cells, T‐lymphocyte activators, parasite‐phagocytic cells, effector cells responsible for parasite elimination, and inducers of immune exhaustion and lymphocyte apoptosis [34]. Although there was a trend toward a reduced number of Iba1^+^ at the end of the treatment, statistical significance was not attained, possibly owing to the short treatment duration and/or the relatively low number of dogs. However, despite the nonsignificant change in the number of these cells, the cutaneous parasitic density was significantly decreased, implicating a lower number of parasite per macrophage. This is a consequence of the direct effect of pentavalent antimonials on macrophages because they increase their phagocytic and parasiticidal activity through priming their respiratory burst [36, 37, 38, 39, 40, 41, 42].

The FoxP3^+^ Treg cells are among the major immune‐regulatory cells essential to dampen excessive inflammatory responses. Although meglumine antimoniate/allopurinol combination, with or without feeding an immunomodulatory diet, resulted in increased percentage of the CD8+ subset of FoxP3^+^ cells in peripheral blood, no similar change was witnessed in the skin in our study [43]. The lack of changes in the Treg cytokines (IL‐10, TGF‐β) in serum was not unexpected because of the contradictory results of published studies on unstimulated serum concentrations of IL‐10 before and after treatment of CanL [44, 45].

Neutrophil elastase, a marker of neutrophilic activation, is a serine protease produced by activated neutrophils. The decrease in such cells in the skin of treated dogs reflects the reduction in neutrophil infiltration correlating with the clinical and parasitological response and the overall decreased cutaneous inflammation. In mice, neutrophil elastase has been associated with killing of *L. major* through a TLR4‐dependent mechanism [44]. Also, like macrophages, meglumine antimoniate increases the parasiticidal activity of neutrophils by upregulating their respiratory burst [40, 41, 42].

The importance of HDP in the progression of *Leishmania* infections is intimately interconnected with the activation of neutrophils and macrophages. In fact, a recent study in mice highlighted the importance of the opsonising effect of defensins on infected neutrophils [8, 9]. Their activity as alarmins and chemoattractants in response to danger signals alerting the adaptive and modulating the innate immune response is essential for parasite control [8, 39]. Previous studies have shown that during leishmaniosis, neutrophils are rapidly mobilised to the inflammatory site to eliminate the pathogen by the production of reactive oxygen species and the release of HDPs and antimicrobial proteases [8, 26, 46]. In the present study, circulating levels of HDPs did not change after treatment. Unfortunately, we were not able to measure their cutaneous expression to better correlate their local expression with the inflammatory response of the skin.

Leptin has been shown to increase proinflammatory cytokine production by macrophages exposed to *Leishmania* spp. and to stimulate phagocytosis of the protozoon [12, 13, 47, 48]. Leptin, secreted by adipocytes and Treg cells, has been suggested as a possible dual marker of CanL severity and prognosis, owing to its role in immune regulation and macrophage activation [12]. Because of its ability to activate naïve T cells while inhibiting memory and Treg cells [27], and to increase the production of IFN‐γ and TNF‐α [47], it was of interest in the present study. The circulatory levels of leptin did not decrease after treatment, which is likely to be a result of the slow and progressive resolution of CanL [12], and, perhaps, to the improved nutritional plane of the dogs, with increased amount of adipose tissue at the end of the treatment period.

One of the aims of the present study was to determine the cutaneous parasitic load before and after treatment. To do so, a *Leishmania*‐specific qPCR was used as a highly sensitive and accurate examination for this purpose [48]. The qPCR data, in agreement with previous studies, showed the efficacy of the meglumine antimoniate in reducing the number of parasites [49]. Additionally, the decrease in parasitic load was accompanied by clinical improvement in the cohort of dogs enrolled in the present study.

The present study has some limitations. These include the relatively low number of dogs enrolled, as well as the lack of evaluation of the systemic, and not only the cutaneous, immune response before and after treatment of CanL. Additionally, the cut‐off of 300 cells counted in the skin samples is not validated and was selected arbitrarily. Also, meglumine antimoniate monotherapy was used instead of the recommended combination of this drug with allopurinol. This was done because the nonspecific anti‐inflammatory action of allopurinol, that has been described in experimental animals and humans (i.e., free radical scavenging, inhibition of IL‐1β and TNF‐a, inhibition of T‐lymphocyte proliferation), may have influenced the results of the study more than its slow‐appearing specific leishmaniostatic activity [50]. Nevertheless, allopurinol administration, at the recommended dose of 10 mg/kg twice daily, was started in all 12 dogs immediately after the end of the study.

In conclusion, this study demonstrates that a complex immune response is occurring in the skin of dogs with CanL and it is modified after short‐term treatment with meglumine antimoniate. In particular, a decrease in T‐bet^+^ (Th1), GATA3^+^ (Th2), IL‐17A^+^ (Th17) cells and neutrophils in response to leishmanicidal treatment was found yet it was not accompanied by similar changes in circulating cytokine concentrations. This study represents a first step toward a better understanding of the cutaneous and systemic immune response before and after treatment of dogs with CanL.

## Author Contributions

**M. Platenik:** methodology, writing – review and editing, data curation, investigation, writing – original draft. **A. Di Loria:** conceptualization, methodology, data curation, supervision, funding acquisition, writing – review and editing, project administration, resources, validation, investigation, visualisation. **L. Archer:** methodology, data curation, writing – review and editing, investigation, software. **M. Saridomichelakis:** writing – review and editing, data curation, validation, conceptualization, methodology, investigation, visualisation, formal analysis. **A. S. Benvenuto:** investigation, methodology. **D. Santoro:** conceptualization, investigation, funding acquisition, writing – review and editing, validation, methodology, visualisation, formal analysis, supervision, data curation, resources.

## Funding

This study was partially funded by the ESVD research grant.

## Disclosure

Artificial Intelligence Generated Content (AIGC) was not used in developing any portion of their manuscript within the Materials and Methods section of their manuscript.

## Conflicts of Interest

The authors declare no conflicts of interest.

## Supporting information


**TABLE S1:** Median counts of cells positive for T‐box transcription factor TBX21 (T‐bet), GATA binding protein 3 (GATA3), interleukin (IL)17‐A, forkhead box protein P3 (FoxP3), ionised calcium‐binding adapter molecule 1 (Iba‐1) and neutrophil elastase before and after 28 days of treatment with meglumine antimoniate (*n* = 12). Dots represent individual data points and horizontal lines indicate the median values.
**TABLE S2:** Concentrations of host defence peptides, leptin and cytokines in the serum before and after 28 days of treatment with meglumine antimoniate (*n* = 12). Dots represent individual data points and horizontal lines indicate the median. Abbreviations: cBD3‐like, canine β‐defensin 3‐like; cCath, canine cathelicidin; IFN, interferon; IL, interleukin; TGF, transforming growth factor.
**TABLE S3:** Quantitative PCR results of *Leishmania* density in the skin before and after 28 days of treatment with meglumine antimoniate (*n* = 6).

## Data Availability

The data that support the findings of this study are available from the corresponding author upon reasonable request.
