# Supplementary material for: Evaluation of the Cutaneous Immunological Milieu Before and After Treatment With Meglumine Antimoniate in Dogs Naturally Affected by Leishmaniosis due to Leishmania infantum
Source: Vet Dermatol. 2026 Feb 18;37(3):455–63. doi: 10.1111/vde.70056 (PMC13167640; doi:10.1111/vde.70056)
Supplement: Supplementary file 1 — TABLE S1: Median counts of cells positive for T‐box transcription factor TBX21 (T‐bet), GATA binding protein 3 (GATA3), interleukin (IL)17‐A, forkhead box protein P3 (FoxP3), ionised calcium‐binding adapter molecule 1 (Iba‐1) and neutrophil elastase before and after 28 days of treatment with meglumine antimoniate (n = 12). Dots represent individual data points and horizontal lines indicate the median values. TABLE S2: Concentrations of host defence peptides, leptin and cytokines in the serum before and after 28 days of treatment with meglumine antimoniate (n = 12). Dots represent individual data points and horizontal lines indicate the median. Abbreviations: cBD3‐like, canine β‐defensin 3‐like; cCath, canine cathelicidin; IFN, interferon; IL, interleukin; TGF, transforming growth factor. TABLE S3: Quantitative PCR results of Leishmania density in the skin before and after 28 days of treatment with meglumine antimoniate (n = 6). [file VDE-37-455-s001.docx]

**TABLE S1.** Median counts of cells positive for T-box transcription factor TBX21 (T-bet), GATA binding protein 3 (GATA3), interleukin (IL)17-A, forkhead box protein P3 (FoxP3), **ionised calcium-binding adapter molecule 1 (**Iba-1) and neutrophil elastase before and after 28 days of treatment with meglumine antimoniate (n = 12). Dots represent individual data points and horizontal lines indicate the median values.

| Markers | Median number of cells (range) (before treatment) | Median number of cells (range) (after treatment) | Median (range) % cell reduction | Significance (*p*-value) |
| --- | --- | --- | --- | --- |
| T-bet | 18.5 (0–300) | 8 (0–100) | 50 | 0.0156 |
| GATA-3 | 87.5 (0–300) | 61.5 (0–300) | 38.9 | 0.0156 |
| IL-17A | 102.5 (0–300) | 33 (0–300) | 56.7 | 0.0313 |
| FoxP3 | 8 (0–300) | 13 (0–122) | 44.7 | 0.0781 |
| Iba-1 | 68.5 (0–300) | 23.5 (0–300) | 65.8 | 0.156 |
| Neutrophil elastase | 50 (0–300) | 11.5 (0–200) | 75.7 | 0.0078 |

**TABLE S2.** Concentrations of host defence peptides, leptin and cytokines in the serum before and after 28 days of treatment with meglumine antimoniate (n = 12). Dots represent individual data points and horizontal lines indicate the median. Abbreviations: cBD3-like, canine β-defensin 3-like; cCath, canine cathelicidin; IFN, interferon; IL, interleukin; TGF, transforming growth factor.

| Markers | Median (range) (before treatment) | Median (range) (after treatment) | Median % reduction | Significance (*p*-value) |
| --- | --- | --- | --- | --- |
| cBD3-like (ng/mL) | 22.5 (6.4–610.1) | 19.1 (6–614.5) | 5.8 | 0.910 |
| cCath (ng/mL) | 8.9 (3.9–20) | 9.2 (4.7–61.4) | –0.3 | 0.569 |
| Leptin (pg/mL) | 2 (0–5.1) | 2.1 (0.5–5.8) | –36.1 | 0.444 |
| IL-4 (pg/mL) | 52 (8.6–2,056.8) | 77.6 (13.9–1,810.6) | –5.1 | 0.622 |
| IL-10 (pg/mL) | 1.92 (0–1,587.8) | 2.67 (0–1,263.6) | 0 | 0.687 |
| IFN-γ (pg/mL) | 10.4 (0–484) | 32.9 (0–550.4) | –33 | 0.492 |
| TGF-β (pg/mL) | 52,028 (16,045.8–70,918.9) | 59,725.5 (19,662.3–86,574.7) | –18.8 | 0.132 |

**TABLE S3.** Quantitative PCR results of *Leishmania* density in the skin before and after 28 days of treatment with meglumine antimoniate (n = 6).

|  | Median (range) (before treatment) | Median (range) (after treatment) | Median % reduction | Significance (*p*-value) |
| --- | --- | --- | --- | --- |
| Parasites / mL | 214 (13–2,012) | 6 (2–63) |  | 0.03 |
